# Protective effects of acute exercise preconditioning on disuse-induced muscular atrophy in aged muscle: a narrative literature review

**DOI:** 10.1186/s12576-020-00783-w

**Published:** 2020-11-27

**Authors:** Toshinori Yoshihara, Hisashi Naito

**Affiliations:** Graduate School of Health and Sports Science, Juntendo University, 1-1 Hirakagakuendai, Inzai, Chiba 270-1695 Japan

**Keywords:** Growth arrest and DNA damage-inducible 45α, Histone deacetylase 4, Exercise intervention, Aged skeletal muscle, Sarcopenia

## Abstract

Aging is associated with a progressive loss of skeletal muscle mass and strength, resulting in frailty and lower quality of life in older individuals. At present, a standard of clinical or pharmacological care to prevent the adverse effects of aging does not exist. Determining the mechanism(s) responsible for muscular atrophy in disused aged muscle is a required key step for the development of effective countermeasures. Studies suggest an age-related differential response of genes and signalings to muscle disuse in both rodents and humans, implying the possibility that effective countermeasures to prevent disuse muscle atrophy may be age-specific. Notably, exercise preconditioning can attenuate disuse-induced muscular atrophy in rodent and human skeletal muscles; however, information on age-specific mechanisms of this exercise-induced protection remains limited. This mini-review aimed to summarize the protective effects of acute exercise preconditioning on muscular atrophy in aged muscle and provide potential mechanisms for its preventive effect on skeletal muscle wasting.

## Background

Aging is associated with frailty, impaired health span, and lower quality of life [1]. Aging impacts muscle adaptations, such as muscle hypertrophy, increased antioxidant capacity, muscle regeneration, and muscle atrophy. Interestingly, several studies have demonstrated that there are age-specific gene and signaling responses to skeletal muscle disuse in both rodents and humans [2–6]. For instance, Leeuwenburgh et al. demonstrated that old rats (32 months old) have a greater apoptotic response to hindlimb unloading in rat soleus muscle than in young rats (6 months old), suggesting that apoptotic regulation during disuse is distinct in young and aged muscles [2]. In human skeletal muscle, one paper comparing age-related differential mechanisms in disuse muscle atrophy (21–27 years vs. 60–72 years) found an age-specific upregulation of Bax and p53 in aged muscle after 2 days of immobility with significant increases in TdT-mediated dUTP nick end labeling and DNA fragmentation in old muscle [6]. Moreover, recent evidence has revealed that growth arrest and DNA damage-inducible 45α (Gadd45α) are required for skeletal muscle atrophy induced by different muscle stressors, such as fasting, denervation, and immobilization [7]. Gadd45α is a soluble, primarily myonuclear protein that causes muscle fiber atrophy by altering skeletal muscle gene expression, stimulating protein breakdown, reducing protein synthesis, decreasing mitochondria, and inhibiting anabolic signaling [7]. It is notable that Gadd45α mRNA expression is significantly increased in the tibialis anterior muscle in 29-month-old rats after 14 days of hindlimb unloading, but not in 9-month-old rats [8]. This suggests that Gadd45α plays a key role in disuse-induced muscle atrophy, especially in aged skeletal muscle. To preserve the functionality of aged muscle, it is important to develop therapeutic countermeasures prior to muscle disuse and identify target molecules that protect against disuse-induced skeletal muscle atrophy.

Although various countermeasures have been developed as potential therapeutic treatments to protect against disuse skeletal muscle atrophy, endurance exercise preconditioning is one of the most practical countermeasures currently available. Even a single bout of preconditioning exercise can attenuate disuse muscle atrophy induced by hindlimb unloading in rats [9]. This evidence suggests that a single bout of preconditioning exercise may be a simple and effective countermeasure against disuse muscle atrophy in aged skeletal muscle. While most older people's circumstances or physical conditions do not permit exercise prior to the atrophic situation, investigating the mechanism(s) responsible for preconditioning exercise-induced protection against disuse muscle atrophy provides unique information to identify biological targets for intervention. By identifying these biological targets, we can develop future therapeutic approaches to prevent muscle wasting. However, at present, there is limited supporting evidence for exercise preconditioning-induced protection against disuse-induced muscle atrophy in aged muscle.

## Differential signaling responses to disuse in aged muscle

Aging affects gene and signaling responses to skeletal muscle disuse in rodents and humans, and investigators have demonstrated that some apoptotic responses to disuse are age-specific [2–6]. Interestingly, recent evidence indicated that growth arrest and DNA damage-inducible 45α (Gadd45α) is required for skeletal muscle atrophy induced by different muscle stressors, such as fasting, denervation, and immobilization [7]. Gadd45α is a soluble, primarily myonuclear protein that alters skeletal muscle gene expression and stimulates protein breakdown, reduces protein synthesis, decreases mitochondria, activates apoptosis, and consequently causes muscle fiber atrophy [7]. Furthermore, histone deacetylase (HDAC) 4, a class II histone deacetylase, is an important regulator of Gadd45α in denervation-induced muscle atrophy [10]. Interestingly, the previous study indicated that Gadd45α mRNA expression was significantly increased in the tibialis anterior muscle in old rats (29 months old) after 14 days of hindlimb unloading, but not in young adult rats (9 months old) [8]. Moreover, Baehr et al. demonstrated that Gadd45α mRNA expression increased significantly after 3 and 7 days of hindlimb unloading in the gastrocnemius muscle and that old rats (29 months old) showed greater Gadd45α mRNA expression for the entire unloading period compared with young adult rats (9 months old) [5]. Activation of these pathways induced the age-related delay in recovery from atrophy [8]. These facts suggest that HDAC4/Gadd45α axis plays an important role in hindlimb unloading-induced muscle atrophy in aged skeletal muscle; thus, HDAC4/Gadd45α axis may be a key pathway for developing potential therapeutic countermeasures in aged muscles before muscle disuse and in identifying target molecules to protect against disuse skeletal muscle atrophy.

## Protective effect of exercise on disuse muscle atrophy in aged muscle

Until now, a variety of countermeasures have been investigated as potential treatments to protect against disuse skeletal muscle atrophy in humans. Physical strategies, such as resistance exercises and maximal voluntary contractions, which can be performed both isometrically and dynamically, are feasible during most immobilization situations and represent powerful tools for preventing muscle atrophy [11]. Moreover, regular exercise can attenuate the major hallmarks of aging, such as genomic instability, loss of proteostasis, mitochondrial dysfunction, cellular senescence, and age-related muscle wasting [12, 13]. Based on previous human studies, exercise, particularly resistance exercise, is a practical countermeasure to age-related muscle atrophy; however, the underlying mechanisms by which exercise preconditioning may prevent adverse effects on aging muscles remain unknown. Notably, even a single bout of preconditioning exercise can attenuate disuse muscle atrophy induced by hindlimb unloading in the rat. For instance, Fujino et al. [9] demonstrated that a bout of exercise preconditioning (20° slope, 20 m/min, 25 min) without pre-familiarization before 2 weeks of hindlimb unloading attenuated slow-type soleus muscle atrophy by preventing mRNA expressions of the proteolytic pathway (cathepsin l, calpain, caspase-3, and E3 ubiquitin ligases) in 9- to 10-week-old male Wistar rats. This suggests that a single bout of preconditioning exercise may effectively suppress the upregulation of protein degradation during disuse in aged skeletal muscle. Additionally, we recently demonstrated that a bout of exercise could suppress Gadd45α upregulation in the gastrocnemius muscle of old rats [14]. As Gadd45α appears to have an age-specific change in response to hindlimb unloading, it is a potential target for exercise-induced protection against disuse muscle atrophy. Moreover, recent work has demonstrated that HDAC4 is an important regulator of Gadd45α in denervation-induced muscle atrophy, and the knockout of HDAC4 can attenuate denervation-induced muscle atrophy [10]. Therefore, exercise preconditioning-induced prevention of Gadd45α via HDAC4 should be effective for aged muscle atrophy. Similarly, we found that acute exercise preconditioning (0° slope, 20 m/min, 15 min) without pre-familiarization can prevent HDAC4 protein and mRNA upregulation in old rats (24-month-old male Wistar rats) with the prevention of downstream Gadd45α [14]. This study's exercise protocol was relatively low-intensity and short-duration compared with the previous study performed by Fujino et al.; therefore, no protective effect was observed in 3-month-old (young) rats.

**Fig. 1:**
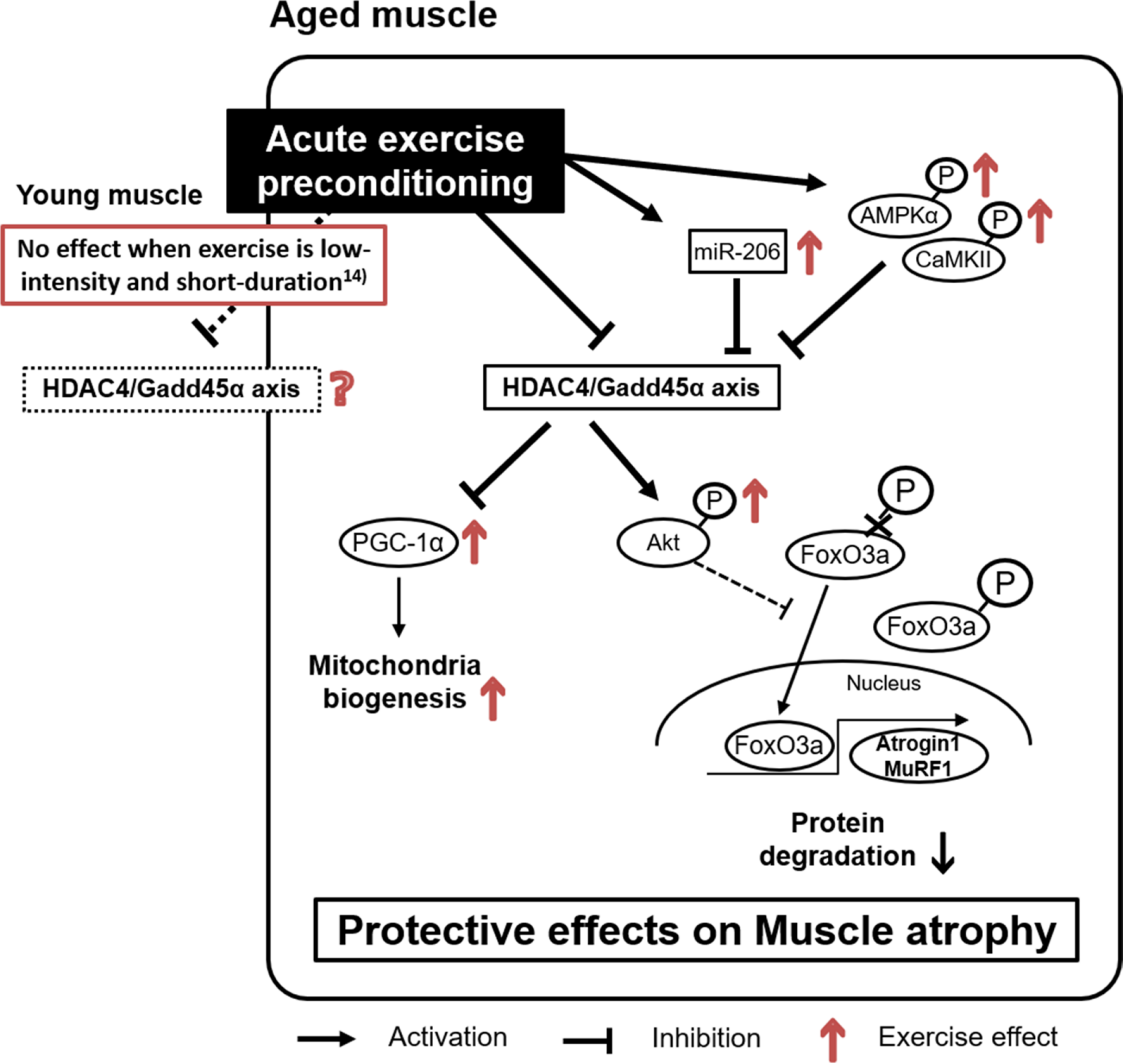
Potential underlying mechanisms of the protective effects of acute exercise preconditioning on disuse-induced muscular atrophy in aged muscle. HDAC4/Gadd45α axis plays an important role in disuse-induced muscle atrophy in aged skeletal muscle via preserving peroxisome proliferator-activated receptor-gamma coactivator-1 (PGC-1α) expression and maintaining Akt phosphorylation during disuse. Other potential mechanisms of exercise-induced protection against the HDAC4/Gadd45α axis include the upregulation of microRNA (miR)-206 and the phosphorylation of AMP-activated protein kinase α (AMPKα) and calcium/calmodulin-dependent kinase II (CaMKII). Arrows represent the exercise effects based on the results from the previous study [14]

Although the precise mechanisms of acute exercise preconditioning-induced protection against muscle atrophy are still unknown, there are some candidate mechanisms that indicate exercise can prevent HDAC4/Gadd45α pathway upregulation in aged muscle (Fig. 1). First, a previous study reported that Gadd45α reduces multiple barriers to muscle atrophy, including peroxisome proliferator-activated receptor-gamma coactivator-1 (PGC-1α) [7]. PGC-1α plays a crucial role in the exercise-induced regulation of muscle atrophy, mitochondrial biogenesis, energy metabolism, and muscle fiber type in old mice skeletal muscle [15]; thus, preserving PGC-1α expression via Gadd45α downregulation seems to be one of the key factors of exercise-induced protection against muscle atrophy. Second, the exercise preconditioning-induced protection against HDAC4/Gadd45α pathway can maintain Akt phosphorylation during disuse [14]. Akt phosphorylation regulates FoxO3a phosphorylation [16, 17], leading to the exclusion of phosphorylated FoxO3a proteins from the nucleus and the inhibition of transcriptional function to induce the downregulation of E3 ligases. Thus, at least in part, it plays an important role in the protective effect on disuse muscle atrophy in old rats [14]. Additionally, microRNA (miR)-206, a member of muscle-enriched miRNAs, is known to facilitate muscle differentiation by regulating the expression of myogenic regulatory factors [18]. Previous studies have reported that miR-206 regulates HDAC4 expression in the muscle under atrophic conditions [18, 19]. Overexpression of miR-206 decreased endogenous HDAC4 levels in the tibialis anterior muscles of mice [20], and miR-206 can attenuate denervation-induced rat skeletal muscle atrophy through the HDAC4-related signaling [21]. In human skeletal muscle, 2 h of acute resistance exercise increased miR-206 [22], and 90 min of ergometer exercise increased miR-206, but load carriage treadmill running did not increase miR-206 [23], suggesting that the effect of acute exercise on miR-206 expression in humans might depend on the exercise conditions. Other potential mechanisms of exercise-induced protection against the HDAC4/Gadd45α axis are the phosphorylation of AMP-activated protein kinase α (AMPKα) and calcium/calmodulin-dependent kinase II (CaMKII) [24]. In disuse conditions, such as limb immobilization, AMPKα and CaMKII are both de-activated and affect HDAC4 phosphorylation during muscle atrophy [25, 26], resulting in upregulation of Gadd45α and E3 ligases. In contrast, exercise preconditioning reverses these effects and prevents HDAC4/Gadd45α pathway upregulation in aged muscle. Nonetheless, the information on exercise-induced protection against disuse muscle atrophy is still limited; therefore, future studies are required to clarify the mechanisms through which exercise prevents muscle atrophy in aged skeletal muscle.

## Conclusions

The evidence suggests that acute exercise preconditioning is an effective countermeasure against a reduction in muscle mass in aged skeletal muscle, and the mechanisms of exercise-induced protection against skeletal muscle loss may be age-specific. Moreover, this protective effect in the aged muscle may be partially mediated by the HDAC4/Gadd45α axis and subsequent protein degradation systems. Currently, supporting evidence in this area is limited, especially regarding the age-specific effects of chronic exercise on disuse-induced muscle atrophy. Therefore, future studies should clarify mechanisms of exercise-induced protection on muscular atrophy in aged muscle and lead to developing therapeutic approaches to prevent muscle wasting.

## Data Availability

Not applicable.

## Abbreviations

Gadd45α: Growth arrest and DNA damage-inducible 45α
HDAC: Histone deacetylase
PGC-1α: Peroxisome proliferator-activated receptor gamma coactivator-1α
miR: MicroRNA
AMPKα: AMP-activated protein kinase α
CaMKII: Calcium/calmodulin-dependent kinase II
